# Low Annexin A1 level in HTLV-1 infected patients is a potential biomarker for the clinical progression and diagnosis of HAM/TSP

**DOI:** 10.1186/s12879-021-05917-y

**Published:** 2021-02-25

**Authors:** Bárbara Brasil Santana, Maria Alice Freitas Queiroz, Rodrigo Arcoverde Cerveira, Claudia Mendonça Rodrigues, Ednelza da Silva Graça Amoras, Carlos Araújo da Costa, Maisa Silva de Sousa, Ricardo Ishak, Luiz Ricardo Goulart, Antonio Carlos Rosário Vallinoto

**Affiliations:** 1 Laboratório de Virologia, Instituto de Ciências Biológica, Universidade Federal do Pará, Belem, 66.075-110 Brazil; 2 Graduate Program in Biology of Infectious and Parasitic Agents, Biological Science Institute, Federal University of Pará, Belem, 66.075-110 Brazil; 3 Laboratory of Nanobiotechnology, Biotechnology Institute, Federal University of Uberlândia, Uberlândia, 38.400-902 Brazil; 4 Laboratory of Cellular and Molecular Biology, Tropical Medicine Center, Federal University of Pará, Belem, 66.055-240 Brazil

**Keywords:** Annexin A1, HTLV, HAM/TSP, Infection, Biomarker

## Abstract

**Background:**

Human T-lymphotropic virus 1 (HTLV-1) is etiologically associated with the chronic inflammatory neurodegenerative disease HTLV-1-associated myelopathy/tropical spastic paraparesis (HAM/TSP) Annexin A1 (AnxA1) is an anti-inflammatory protein with proposed neuroprotective and anti-neuroinflammatory functions. We hypothesized that *ANXA1* gene expression may be dysregulated in HTLV-1-infected HAM/TSP patients.

**Methods:**

This study involved 37 individuals infected with HTLV-1, including 21 asymptomatic (AS) carriers and 16 with HAM/TSP, and a control group of 30 individuals negative for HTLV-1 and HTLV-2. For AS HTLV-1-positive and HAM/TSP patients, *ANXA1* and formyl peptide receptor (*FPR1*, *FPR2* and *FPR3*) expression and HTLV-1 proviral load (PVL) in peripheral blood cells were evaluated by real-time quantitative PCR (qPCR), and plasma AnxA1 levels were determined by enzyme-linked immunosorbent assay (ELISA).

**Results:**

*ANXA1* gene expression was increased in the AS group compared with the HAM/TSP and control groups, but the differences were not statistically significant. *FPR1* gene expression was higher in patients with HTLV-1 than in controls (AS, *p* = 0.0032; HAM/TSP, *p* < 0.0001). Plasma AnxA1 levels were higher in the AS group than in the HAM/TSP group (*p* = 0.0045), and PVL was higher in patients with HAM/TSP than in AS individuals (*p* = 0.0162). The use of a combined ROC curve using Annexin 1 levels and proviral load significantly increased the sensitivity and specificity to predict progression to HAM/TSP (AUC = 0.851 and AUC = 0.937, respectively, to AUC = 1000).

**Conclusions:**

Our results suggest that AnxA1 may be dysregulated in HAM/TSP patients. Serological detection of AnxA1 in association with proviral load may provide a prognostic biomarker for HTLV-1-associated neurodegenerative disease.

**Supplementary Information:**

The online version contains supplementary material available at 10.1186/s12879-021-05917-y.

## Background

Human T-lymphotropic virus 1 (HTLV-1) is a member of the family *Retroviridae*, subfamily *Orthoretrovirinae*, genus *Deltaretrovirus* [1] that is endemic in Japan, the Caribbean, South America, Sub-Saharan Africa and Melanesia [2]. HTLV-1 infection is associated with adult T cell leukemia/lymphoma (ATLL), mature CD4^+^ T cell neoplasms, and HTLV-1-associated myelopathy/tropical spastic paraparesis (HAM/TSP), a chronic and progressive neurodegenerative disease [3, 4].

HAM/TSP is a chronic, progressive, demyelinating disease that affects the spinal cord and brain white matter, leading to the onset of a severe clinical syndrome involving motor impairment of the lower limbs [4]. The clinical picture begins and evolves insidiously, and it is often impossible to establish the initial onset of symptoms [5].

Several studies have identified Tax regulatory protein as the main target of the immune response against HTLV-1, as this antigen is most efficiently recognized by cytotoxic T lymphocytes [6–9]. HTLV-1 infection induces the activation and robust proliferation of infected T lymphocytes. This phenomenon is mainly related to the function of the viral *tax* gene, which is involved in transactivation of the interleukin-2 (IL-2) and IL-2 receptor genes, among others [10]. Indiscriminate cell proliferation can also lead to the expansion of self-reactive T cells and the marked secretion of proinflammatory cytokines, such as tumor necrosis factor alpha (TNF-α). These abnormalities are associated with the neurological damage observed in patients with HAM/TSP [11].

Based on the recognition that a sustained systemic inflammatory response contributes to chronic neurodegenerative disorders, we hypothesized that the anti-inflammatory response may be dysfunctional in HAM/TSP patients. Among many anti-inflammatory molecules, annexin A1 (AnxA1) likely plays an important role in modulating neuroinflammation triggered by both resident glial cells as part of the innate immune system in the brain and circulating leukocytes that breach the blood–brain barrier [12]. AnxA1, a 37-kDa protein that belongs to the annexin superfamily [13, 14], has been identified as a glucocorticoid-induced anti-inflammatory protein involved in eicosanoid and phospholipase A2 synthesis [15, 16]. The secretion of AnxA1 is key for anti-inflammatory activity, as this protein binds in an autocrine or paracrine manner to specific receptors on the outer leaflet of the plasma membrane of target cells, reducing proinflammatory activity [17–19]. AnxA1 receptor (formyl peptide receptor: FPR1, FPR2 and FPR3) expression is particularly high on the plasma membranes of macrophages, monocytes and neutrophils [19–22].

Thus, considering that HAM/TSP is a systemic immune disorder caused by Th1 cell activation and increased levels of proinflammatory cytokines, we hypothesized that AnxA1 is dysfunctional in this context, which may impact its neuroprotective immunomodulatory role. Therefore, the present work investigated the associations of *ANXA1* gene expression profiles and protein levels with the development of HAM/TSP.

## Methods

### Case sample

A total of 57 individuals participated in this study and were divided into the following groups:

Group 1 (asymptomatic, AS): 21 AS HTLV-1 carriers who were positive by enzyme-linked immunosorbent assay (ELISA) and real-time quantitative polymerase chain reaction (qPCR).

Group 2 (HAM/TSP): 16 HTLV-1 carriers who were by positive ELISA and real-time PCR with a clinically confirmed HAM/TSP diagnosis.

Group 3 (control group, CG): 20 individuals negative for HTLV-1 and HTLV-2 by serological and molecular tests.

The clinical classification of patients was performed by a neurologist at the Tropical Medicine Center (NMT) in Belém, State of Pará, Brazil, following the protocol of De Castro-Costa et al. [23]. The main clinical symptoms diagnosed in the HAM/TSP group were low back pain, constipation, leg weakness, increased deep reflexes, bladder disturbance, cramps and Babinski’s sign. Blood samples were collected between August 2015 and May 2017 at the NMT, and laboratory procedures were conducted at the Laboratory of Virology (LabVir) of the Federal University of Pará (UFPA). Blood samples (5 mL) obtained by venipuncture were placed in 2 vacutainers containing ethylenediaminetetraacetic acid (EDTA) as an anticoagulant and were used for flow cytometry, ribonucleic acid (RNA) extraction and plasma AnxA1 measurements. All subjects were seronegative for human immunodeficiency virus 1 (HIV-1). HAM/TSP patients were not on anti-inflammatory treatment at the time of the study.

### RNA extraction, quantification and reverse transcription

RNA was extracted from whole blood cells using TRIzol® reagent (Applied Biosystems, Foster City, CA, USA) following the manufacturer’s protocol. RNA was quantified in the SpectraMax® i3 Multi-Mode Detection Platform, which uses a 24-well microplate containing 2 μL of elution buffer in one well as a blank and 2 μL of each sample in the other wells. The absorbance of the samples was read at a wavelength of 260 nm. Then, 20 ng of deoxyribonuclease I (DNase I)-treated RNA was used for the reverse transcription of messenger RNA (mRNA) into complementary DNA (cDNA) using the High Capacity cDNA Reverse Transcription kit (Applied Biosystems, USA), following the manufacturer’s technical recommendations. The cDNA was stored at − 20 °C prior to use.

### *ANXA1*, *FPR1*, *FPR2* and *FPR3* gene expression

cDNA was analyzed using real-time quantitative PCR (relative quantification (RQ) by the ΔΔCT method). The qPCR results for endogenous genes and targets were standardized to calculate the efficiency of the amplification reactions. Different concentrations of cDNA were tested (undiluted and 4 serial dilutions using a factor of 2, from 1:2 to 1:16). Reactions were performed in triplicate wells and analyzed simultaneously using the same cDNA (at different dilutions) with different probes to construct an efficiency curve and validate the 2^-ΔΔCT^ analysis method. All assays showed the expected efficiency (100 ± 10%; Supplementary Fig. 1). The RQ of target gene expression was conducted based on the comparative CT method (ΔΔCT) using the 2^-ΔΔCT^ formula, where ΔΔCT = ΔCT_sample_-ΔCT_reference_ [24]. *ANXA1*, *FPR1*, *FPR2* and *FPR3* mRNA levels were quantified using GoTaq Green Master Mix (Promega, Madison, WI, USA) with *β-actin* as the reference gene. The reactions were carried out in the StepOne PLUS Sequence Detector (Applied Biosystems, Foster City, CA, USA). The primer sequences are provided in Table 1. The reactions included 1X GoTaq Green Master Mix [2X], 0.5 pmol/μL primer [10 pmol/μL] and 60 ng of cDNA in a final reaction volume of 20 μL. The temperature conditions were as follows: 95 °C (hold stage) for 20 s, followed by 40 cycles of 95 °C (denaturation) for 15 s and 60 °C (primer binding and product extension) for 20 s. The melting curves for all the samples were evaluated after the reaction ended, and those for the investigated genes are presented in Supplementary Fig. 2.

**Table 1:** Nucleotide sequences of the primers used for real-time PCR to quantify *ANXA1*, *FPR1*, *FPR2* and *FPR3* mRNA levels

Primer	Sequence (5′-3′)	Direction
ANXA1F	GATTTTCGGAACGCTTTGCT	Forward
ANXA1R	AGTCCTCAGATCGGTCACCCT	Reverse
FPR1F	ACCCAGAGCAAGACCACAGC	Forward
FPR1R	TCCATCTTGTCTGCTCCTGCA	Reverse
FPR2F	ATTTGCAGCCTTGAGGTCA	Forward
FPR2R	AGCACCTGGTGCATTTTCCT	Reverse
FPR3F	GGATGACACGCACAGTCAACA	Forward
FPR3R	TCAGCTAGGGCCAGGTTCAG	Reverse
BACTINAF	TCCCTGGAGAAGAGCTACG	Forward
BACTINAR	TAGTTTCGTGGATGCCACA	Reverse

### Quantification of plasma AnxA1 levels

Plasma AnxA1 levels were measured by ELISA (Human Annexin A1 ELISA Kit, ab222868, Abcam, Cambridge, UK) with specific polyclonal anti-human AnxA1 antibodies. The assays were conducted according to the manufacturer’s recommendations.

### Quantification of HTLV-1 proviral load

Proviral load (PVL) was quantified by qPCR using three target sequences synthesized using the TaqMan® system (Life Technologies, Foster City, CA, USA) according to a previously described protocol [25]. The results were adjusted to obtain the absolute proviral quantification considering the leukocyte count per mm^3^, and the final results are presented as DNA proviral copies/mm^3^.

### Statistical analysis

Normality analysis of the sample distribution was performed using the Kolmogorov-Smirnov test. Target gene expression levels and the percentages of *ANXA1*-expressing immunoinflammatory cells were compared among groups using the nonparametric Kruskal-Wallis test. Significant results in the Kruskal-Wallis test were subjected to multiple comparisons analysis by Dunn’s post test. Plasma AnxA1 levels and PVL were compared between the HAM/TSP and AS groups by the Mann-Whitney test. Receiver operating characteristic (ROC) curves were made to investigate diagnostic accuracy in the PVL, AnxA1 and PVL + AnxA1 tests in relation to sensitivity and specificity. The area under the ROC curve (AUC) represents the ability of the test to correctly classify participants with HAM/TSP and progression to disease. The AUC values vary between 1 (diagnosis correctness) and 0 (diagnosis error). The tests were performed using BioEstat 5.3 software [26] and the ROC curve analyzes were performed by the programs GraphPad prism 6.0 and SSP 25.0. The results with *p* < 0.05 were considered significant.

## Results

**Fig. 1:**
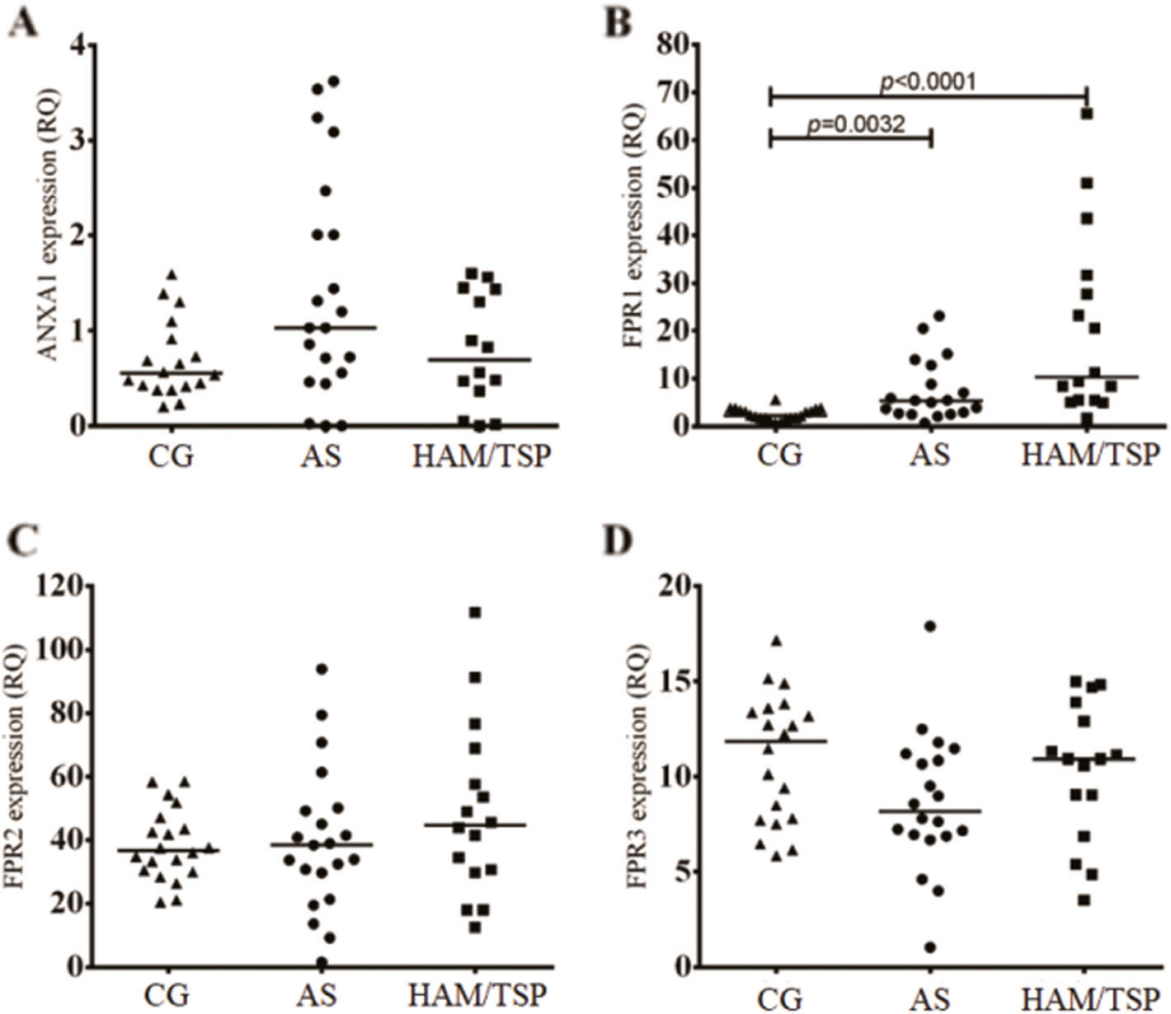
Quantification of (**a**) *ANXA1*, (**b**) *FPR1*, (**c**) *FPR2* and (**d**) *FPR3* mRNA levels in whole blood from the control group (CG), asymptomatic (AS) patients and patients diagnosed with HTLV-1-associated myelopathy/tropical spastic paraparesis (HAM/TSP). RQ: relative quantification. *Median (Kruskal-Wallis test)

Standard curves generated to calculate the amplification efficiency and melting curves for the target genes are shown in Figs. S1 and S2, respectively. Quantification of *ANXA1*, *FPR1*, *FPR2* and *FPR3* gene expression levels in the investigated groups showed lower *ANXA1* mRNA levels in the controls than in the HTLV-1-infected individuals, but these differences were not significant. Among the infected patients, those with HAM/TSP expressed lower *ANXA1* levels (Fig. 1a). The mean Ct values for the reference and target genes in each group are shown in Table S1.

*FPR1* gene expression levels were significantly lower in the control individuals than in those infected with HTLV-1 (Fig. 1b). *FPR2* and *FPR3* expression levels were not different among the groups (Fig. 1 c and d).

**Fig. 2:**
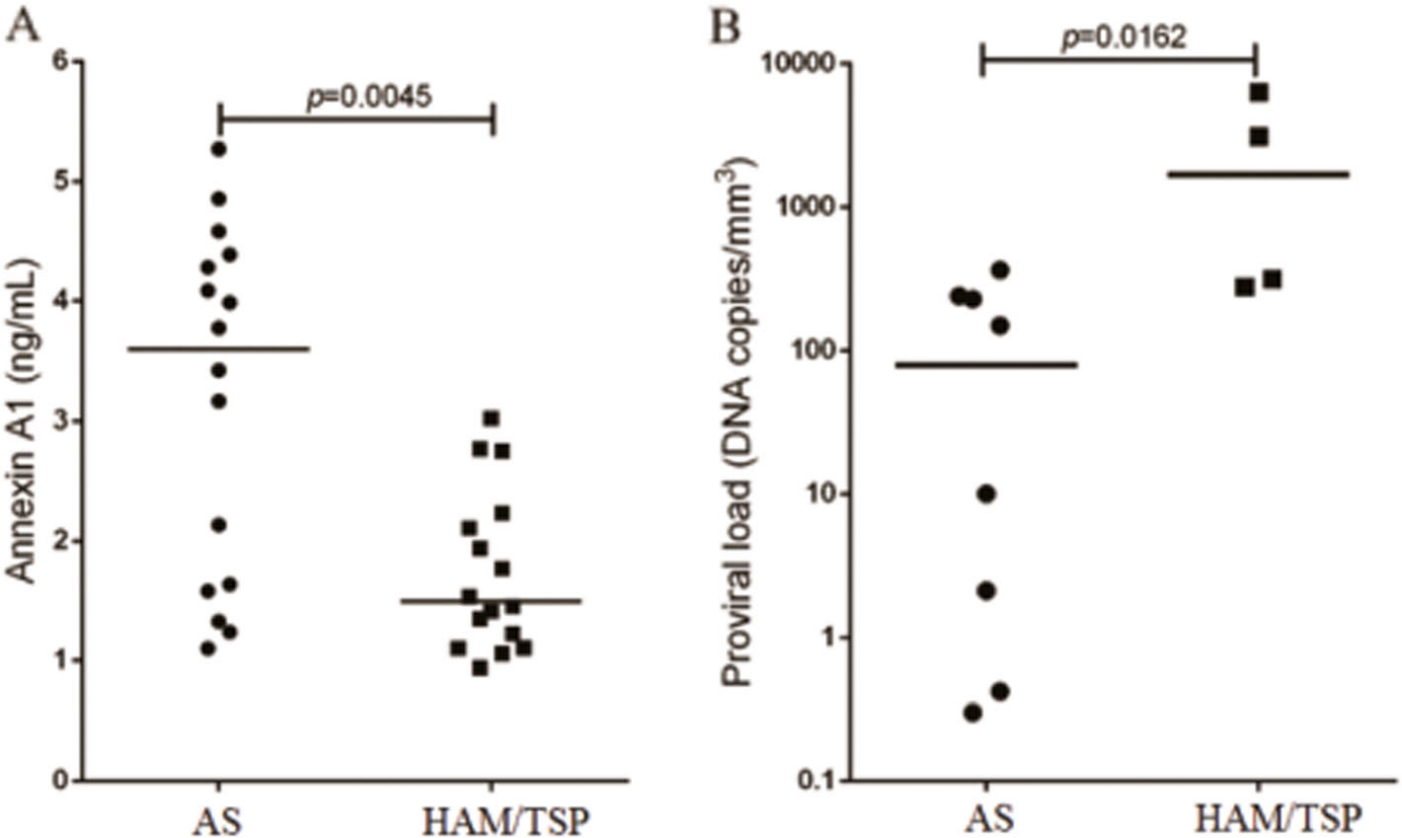
Quantification of (**a**) annexin A1 (AnxA1) plasma levels and (**b**) human T-lymphotropic virus 1 (HTLV-1) proviral load in asymptomatic (AS) patients and patients diagnosed with HTLV-1-associated myelopathy/tropical spastic paraparesis (HAM/TSP). *Median (Mann-Whitney test)

Serum AnxA1 levels were evaluated to assess whether the observed mRNA expression profiles reflect the amount of free AnxA1 in plasma. The AS group had significantly higher serum AnxA1 levels than the HAM/TSP group (*p* = 0.0045, Fig. 2a). In contrast, the PVL was significantly higher (*p* = 0.0162) in the HAM/TSP patients than the AS individuals (Fig. 2b).

**Fig. 3:**
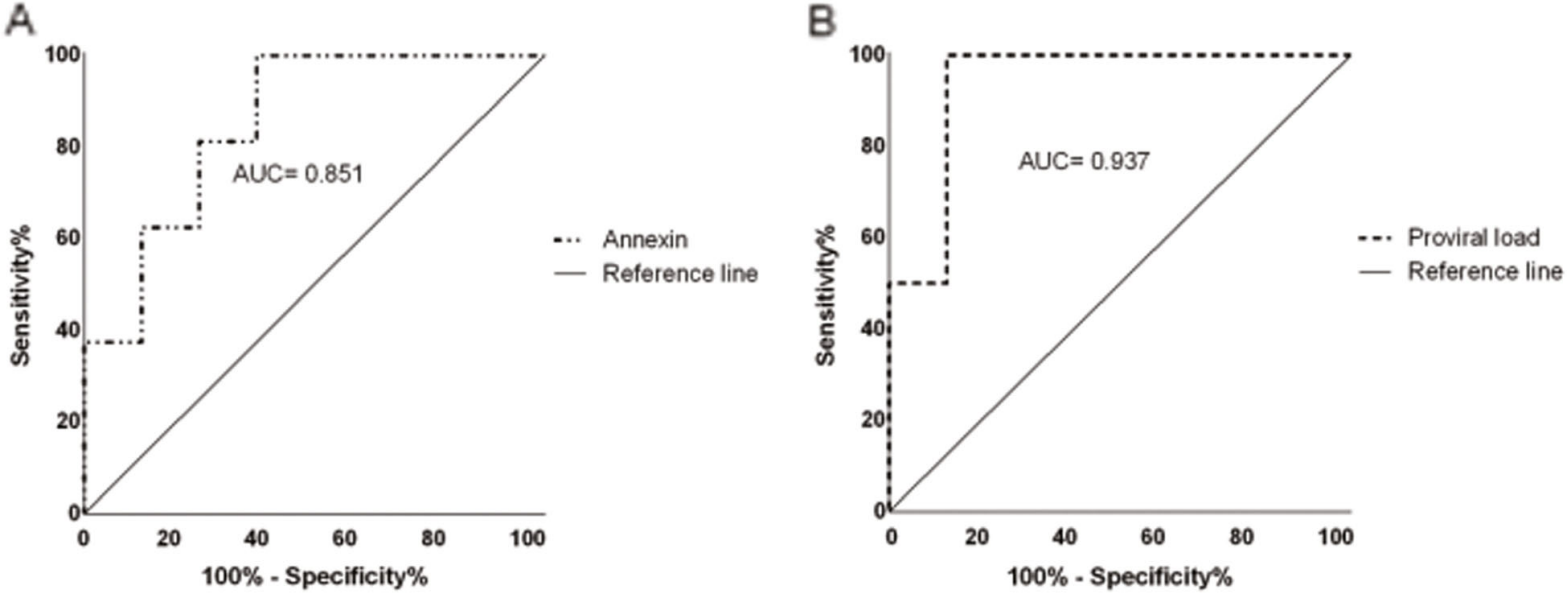
ROC curve of (**a**) Annexin A1 and (**b**) Proviral Load

The levels of Annexin A1 and proviral load were evaluated using the ROC curve to identify the potential of these markers as adjunct laboratory diagnostics for the identification of patients with HAM/TSP. Table 2 shows that the two tests had an area on the curve (AUC) of 0.8516 and 0.9375, respectively. The sensitivity of both fixed at 100% allowed to reach specificity levels of 62.5 and 87.5%, respectively. The value of the best cut-off point for the identification of patients with HAM/TSP was < 3095 for annexin A1 and >  259.5 for proviral load. The best points of the curves are shown in Fig. 3 a and b.

**Table 2:** Evaluation of the sensitivity and specificity of the dosages of Annexin and Proviral Load as biomarkers in the prognosis of HTLV-1 infection

Variable	AUC	***P*** value	***Cut-off***	Sensitivity % (CI%)	Specificity % (CI%)	LR
Annexin A1	0.851	0.0006	< 3.095	100 (79.41–100)	62.50 (35.43–84.80)	2.667
Proviral load	0.937	0.0174	> 259.5	100 (39.76–100)	87.50 (43.75–99.68)	8.000

*AUC* Area under the ROC Curve, *LR* Likelihood ratio

**Fig. 4:**
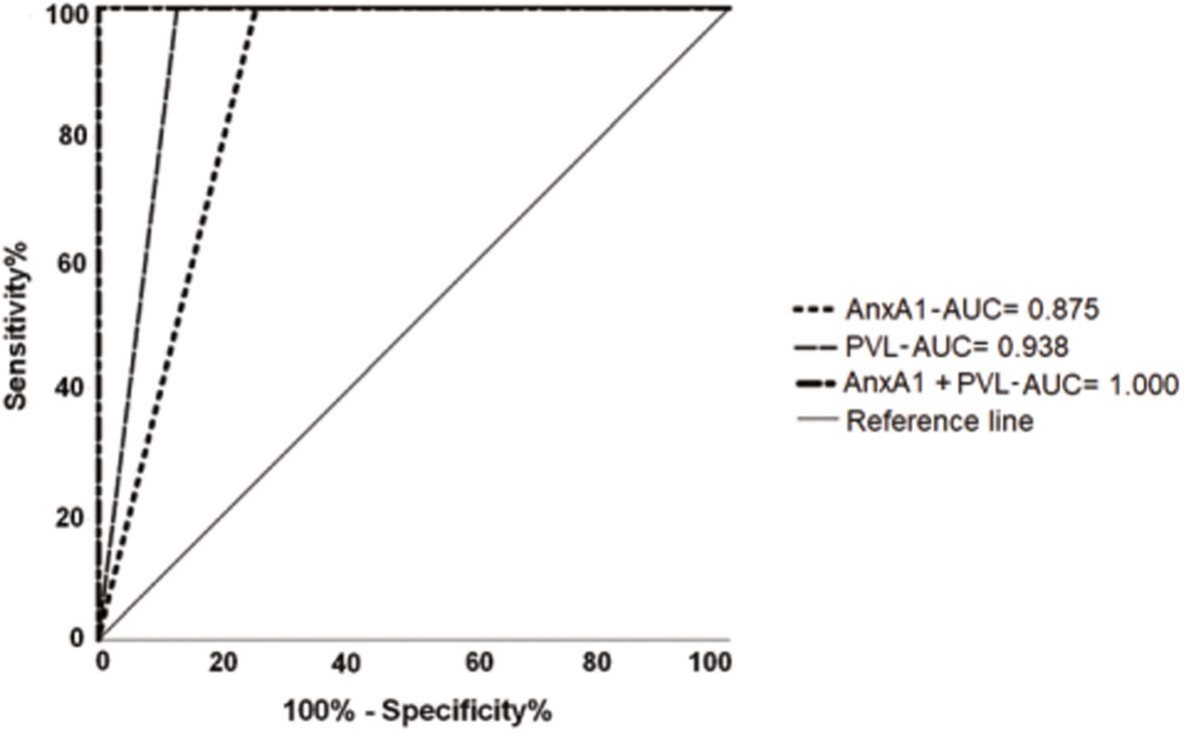
ROC curve of the Annexin A1 and Proviral Load markers used simultaneously to predict progression and diagnostic confirmation of HAM/TSP.

The use of the ROC curve principle, combining the two variables, in order to improve the prediction of asymptomatic progression to HAM/TSP, showed that sensitivity and specificity reached high levels, close to 100% (Table 3). Table S2 show the list of HTLV-1 infected subjects and their Annexin A1 and proviral load levels. Figure 4 shows the representation of the performance of the investigated markers.

**Table 3:** Assessment of Annexin and Proviral Load used simultaneously to estimate the progression and diagnostic confirmation of HAM/TSP.

Test	AUC	***P*** value	Sensitivity %	Specificity %
Annexin A1 (AnxA1)	0.875	0.042	100	75
Proviral load (PL)	0.938	0.017	100	87.50
AnxA1 + PL	1.000	0.007	100	100

*AUC* Area under the ROC Curve

## Discussion

The molecular mechanisms underlying the progressive neurodegeneration that characterizes the pathogenesis of HAM/TSP in HTLV-1-infected patients continue to be discussed by researchers. In this investigation, we suggest an association between the reduced *ANXA1* expression with the possible lack of an anti-inflammatory response in HAM/TSP HTLV-1-infected patients, who are characterized by a sustained systemic inflammatory response and a high PVL that contribute to chronic neurodegenerative disease.

The host genotype (determined mainly by the human leukocyte antigen (HLA) class I and killer cell immunoglobulin-like receptor (KIR) loci), quality of the cytotoxic T cell (CTL) response against HTLV-1-infected cells, T regulatory cell (Treg) frequency (forkhead box protein 3 (FOXP3)^+^) and PVL are important risk factors for the development of TSP/HAM [27–29]. An important prognostic factor for the development of HTLV-1-associated diseases is the PVL in blood as assessed by qPCR [30, 31]. AS carriers tend to have a lower PVL than those who develop HAM/TSP [32, 33].

Given the debate regarding the factors that affect HAM/TSP pathogenesis, in the present study, we investigated the potential association of AS HTLV-1 infection or infection with HAM/TSP symptoms and the gene expression of *ANXA1* and its receptors (FPRs) in peripheral blood cells. The results showed that AS individuals had higher *ANXA1* mRNA levels, which might suggest that AnxA1 is an important factor controlling the inflammatory response that triggers HTLV-1-associated neurodegenerative disease. A previous study suggested that AnxA1 is a potential biomarker for the advanced stage of cell transformation, which is directly linked to the uncontrolled growth of infected cells, accumulation of genetic defects and development of symptoms of HTLV-1-associated diseases [34]. However, the pathophysiology of HAM/TSP has an inflammatory nature that is different from HTLV-1-induced cell transformation, and therefore, it would not be hard to assume that the high endogenous production of the anti-inflammatory protein AnxA1 could have a protective effect on the progression of HAM/TSP.

In addition to the association between *ANXA1* mRNA transcription and HTLV-1 infection, a relationship was observed between infection and *FPR* mRNA levels. The interactions of these receptors with other ligands (both anti- and proinflammatory) enable the regulation of several pathological conditions, including tumorigenesis [35, 36], inflammation [37] and some infectious processes [38].

We observed that the HTLV-1 carriers with HAM/TSP symptoms had higher *FPR1* mRNA levels than HTLV-1-infected AS subjects and controls. Although these data suggest a relationship between *FPR1* and the pathogenesis of HAM/TSP in HTLV-1-infected patients, the evidence remains preliminary, and further mechanistic studies are necessary. Additional studies must be performed to confirm whether the higher viral load somehow triggers increased *FPR1* levels through another receptor or if the lower AnxA1 levels induce *FPR1* expression.

We evaluated serum AnxA1 levels to assess whether the mRNA expression profile reflects free annexin levels in plasma. The quantification showed high AnxA1 plasma levels in AS patients than in HAM/TSP patients, inverse to the PVL results. Although a direct correlation between annexin and PVL (data not shown) was not observed in the present study, AnxA1 seems to be a potential new biomarker that, in addition to PVL, could contribute to the follow-up of HTLV-1-infected subjects, but longitudinal data are required among people with asymptomatic HTLV-1 infection to confirm our hypothesis.

Although the study was cross-sectional and does not do a mechanistic analysis, the results seem to suggest an anti-inflammatory role of AnxA1 in HTLV-1 infection, what if confirmed, could be neuroprotective, since patients with higher levels of this protein could better control the development of HAM/TSP. Thus, the possible antiviral activity of AnxA1 against HTLV-1 must be more thoroughly investigated.

## Conclusions

In conclusion, taken together the quantification of Anexin and proviral load quantification, could be an important laboratory tool to aid in the diagnosis of HAM/TSP. Even though the sample used herein was small, which is always a big problem in the study of HTLV-1, the periodic clinical assessment of HTLV-1 carriers associated with the quantitative analysis of Annexin and proviral load, as used in the present study, seems to be of relevance in the prognostic prediction of the progression of asymptomatic infection to HAM/TSP, considering the high sensitivity and specificity values ​​found. However, we highlight the need of population-based and longitudinal cohort studies aiming to define cut-offs for serum AnxA1 levels among different groups as differential diagnostic criteria and so to confirm our hypothesis.

## Supplementary Information

**Additional file 1.**

## Data Availability

The datasets in this study are available from the corresponding author on reasonable request.

## Abbreviations

HTLV-1: Human T-Lymphotropic virus 1 (HTLV-1)
HAM/TSP: HTLV-1-associated myelopathy/tropical spastic paraparesis;
AnxA1: Annexin A1
PVL: Proviral load
FPR: Formyl peptide receptor
ELISA: Enzyme-linked immunosorbent assay
qPCR: Real-time quantitative polymerase chain reaction
ATLL: Adult T cell leukemia/lymphoma
IL-2: Interleukin-2
TNF-α: Tumor necrosis factor alpha
AS: Asymptomatic
CG: Control group
NMT: Tropical Medicine Center
LabVir: Laboratory of Virology
DNAse: Deoxyribonuclease
cDNA: Complementary DNA
EDTA: Ethylenediaminetetraacetic acid
RNA: Ribonucleic acid
mRNA: Messenger ribonucleic acid
HIV-1: Human immunodeficiency virus 1
HLA: Human leukocyte antigen
KIR: Killer cell immunoglobulin-like receptor
CTL: Cytotoxic T cell
Treg: T regulatory cell
FOXP3: Forkhead box protein 3
