# Supplementary material for: Low Annexin A1 level in HTLV-1 infected patients is a potential biomarker for the clinical progression and diagnosis of HAM/TSP
Source: BMC Infect Dis. 2021 Feb 25;21:219. doi: 10.1186/s12879-021-05917-y (PMC7908672; doi:10.1186/s12879-021-05917-y)
Supplement: Supplementary file 1 — Additional file 1. [file 12879_2021_5917_MOESM1_ESM.zip › Supplementary file.docx]

**Supplementary files**

Title: Low Annexin A1 level in HTLV-1 infected patients is a potential biomarker for the clinical progression and diagnosis of HAM/TSP

Bárbara Brasil Santana^1,4†^, Maria Alice Freitas Queiroz^1†^, Rodrigo Arcoverde Cerveira^1^, Claudia Mendonça Rodrigues^3^, Ednelza da Silva Graça Amoras^1^, Carlos Araújo da Costa^2^, Maisa Silva de Sousa^2^, Ricardo Ishak^1^, Luiz Ricardo Goulart^3^, Antonio Carlos Rosário Vallinoto^1*^

**Figure Supplementary 1.** Standard curves generated to calculate the efficiency of (A) *ANXA1*, (B) *FPR1*, (C) *FPR2*, (D) *FPR3* and (E) *β-actin* amplification.

**Figure Supplementary 2.** Melting curves for (A) *ANXA1*, (B) *FPR1*, (C) *FPR2*, (D) *FPR3* and (E) *β-actin*.

**Table Supplementary 1.** Mean Ct values of the reference and target genes in each group.

| Group | ***FPR1*** | ***FPR2*** | ***FPR3*** | ***ANXA1*** | ***β-actin*** |
| --- | --- | --- | --- | --- | --- |
| Asymptomatic | 28.191 | 22.958 | 19.202 | 26.415 | 18.663 |
| TSP/HAM | 27.918 | 23.248 | 19.351 | 26.659 | 18.698 |
| Control | 31.709 | 23.016 | 21.026 | 27.824 | 17.573 |

Table Supplementary 2. Plasma levels of Annexin A1 and proviral load of the patient evaluated in the study.

| Individuals | Annexin A1 (ng/mL) | Proviral load  (DNA copies/mm3) |
| --- | --- | --- |
| Asymptomatic |  |  |
| #17217 | 1.6386 | * |
| #17218 | 1.5826 | 0.42 |
| #17219 | 2.1346 | * |
| #17221 | 4.3906 | 0.3 |
| #17285 | 4.8566 | * |
| #17416 | 1.2386 | 228.54 |
| #17417 | 1.3266 | * |
| #18378 | 5.2706 | 149.04 |
| #18462 | 4.2846 | * |
| #18463 | 4.5846 | 10.07 |
| #18501 | 3.9886 | * |
| #18502 | 3.7766 | * |
| #18745 | 4.0886 | 240.48 |
| #18856 | 3.1646 | 364.47 |
| #18857 | 3.4226 | 2.12 |
| #21955 | 1.1026 | * |
|  |  |  |
| HAM/TSP |  |  |
| #12823 | 1.5346 | 314.92 |
| #15289 | 1.1046 | 6296.6 |
| #15416 | 1.1066 | 278.44 |
| #19941 | 2.1106 | 3094.18 |
| #21954 | 1.9386 | * |
| #22305 | 1.2266 | * |
| #22443 | 1.3486 | * |
| #25696 | 1.4126 | * |
| #26110 | 0.9406 | * |
| #26112 | 2.7706 | * |
| #26114 | 3.0246 | * |
| #26115 | 2.7546 | * |
| #26116 | 2.2326 | * |
| #26120 | 1.0586 | * |
| #26121 | 1.4526 | * |
| #26122 | 1.7686 | * |

* Not done
