# Supplementary figures and images for: Low Annexin A1 level in HTLV-1 infected patients is a potential biomarker for the clinical progression and diagnosis of HAM/TSP
Source: BMC Infect Dis. 2021 Feb 25;21:219. doi: 10.1186/s12879-021-05917-y (PMC7908672; doi:10.1186/s12879-021-05917-y)

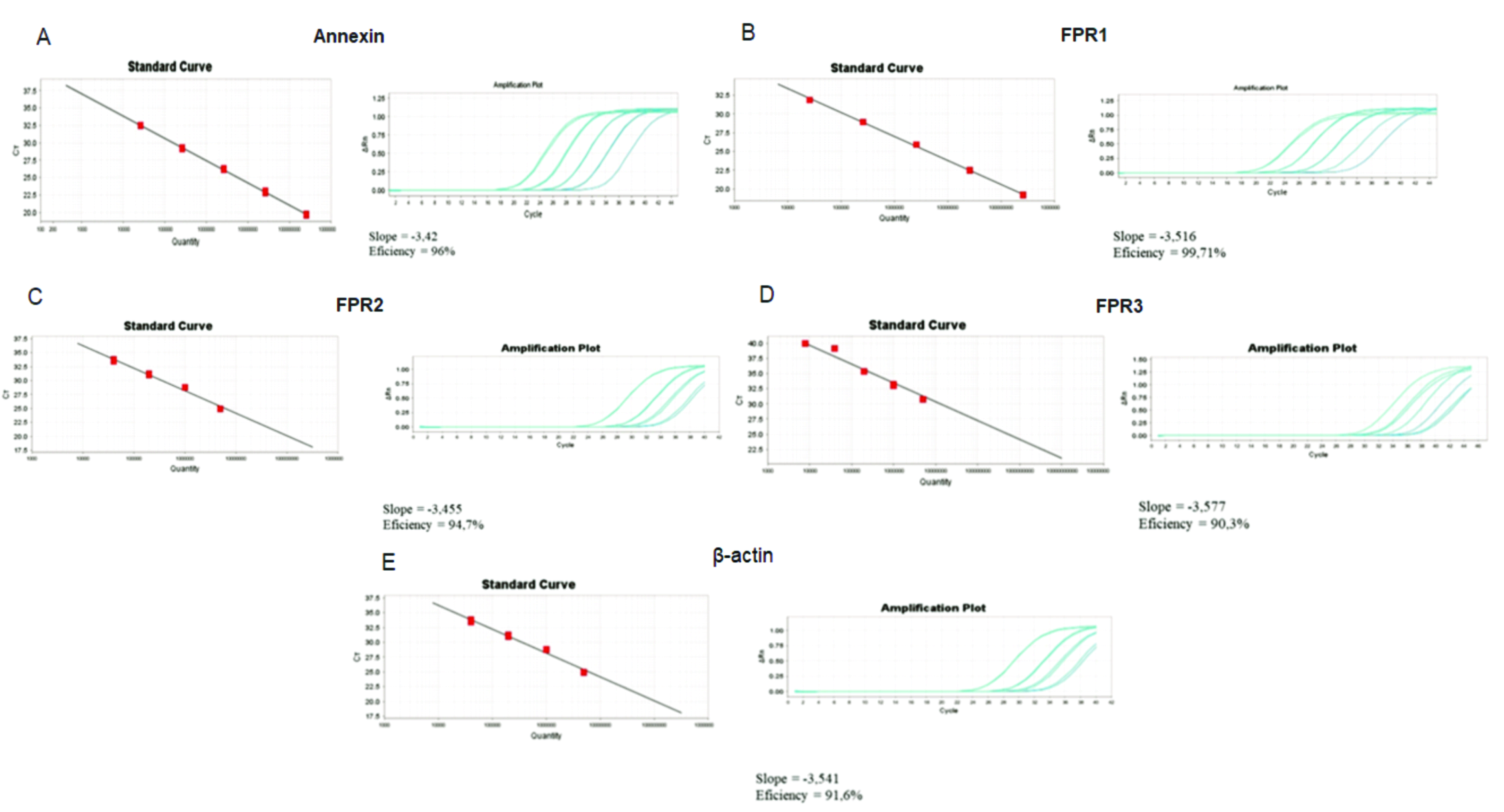

Supplement: Supplementary file 1 — Additional file 1. [file 12879_2021_5917_MOESM1_ESM.zip › Supplementary Figure 1.tif]

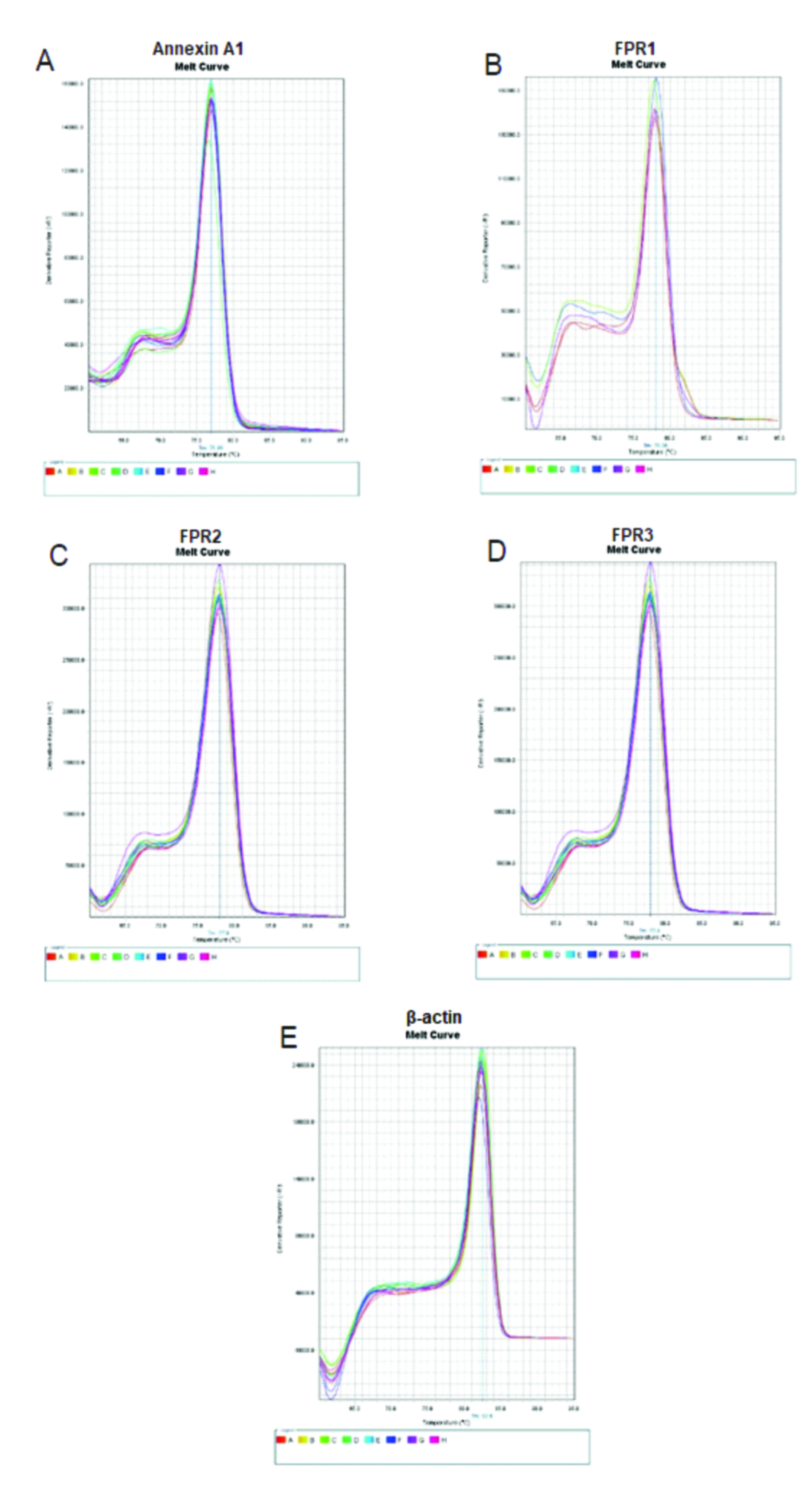

Supplement: Supplementary file 1 — Additional file 1. [file 12879_2021_5917_MOESM1_ESM.zip › Supplementary Figure 2.tif]
